# Evolutionary Invasion Analysis of Modern Epidemics Highlights the Context-Dependence of Virulence Evolution

**DOI:** 10.1007/s11538-024-01313-0

**Published:** 2024-06-14

**Authors:** Sudam Surasinghe, Ketty Kabengele, Paul E. Turner, C. Brandon Ogbunugafor

**Affiliations:** 1 Department of Ecology and Evolutionary Biology, Yale University, New Haven, CT 06520 USA; 2 Public Health Modeling Unit, Yale School of Public Health, New Haven, CT 06510 USA; 3 Microbiology Program, Yale School of Medicine, New Haven, CT 06510 USA; 4 Santa Fe Institute, Santa Fe, NM 87501 USA

**Keywords:** Virulence, Evolutionary stable strategy, Evolutionary invasion analysis, Disease dynamics, Virus evolution

## Abstract

Models are often employed to integrate knowledge about epidemics across scales and simulate disease dynamics. While these approaches have played a central role in studying the mechanics underlying epidemics, we lack ways to reliably predict how the relationship between virulence (the harm to hosts caused by an infection) and transmission will evolve in certain virus-host contexts. In this study, we invoke evolutionary invasion analysis—a method used to identify the evolution of uninvadable strategies in dynamical systems—to examine how the virulence-transmission dichotomy can evolve in models of virus infections defined by different natural histories. We reveal peculiar patterns of virulence evolution between epidemics with different disease natural histories (SARS-CoV-2 and hepatitis C virus). We discuss the findings with regards to the public health implications of predicting virus evolution, and in broader theoretical canon involving virulence evolution in host-parasite systems.

## Introduction

Recent events have reinvigorated interest in the evolution and ecology of infectious disease, specifically, what rules (if any) govern how lethal a given pathogen will become in a population of hosts. These questions have formed a theoretical canon defined by hundreds of studies and analytical descriptions of the evolvability and constraints surrounding how a pathogen evolves increased virulence (Anderson and May 1982; Ewald 1983, 2004; Bull 1994; Lenski and May 1994; Frank 1996; Ebert and Weisser 1997. Virulence can be defined in many ways, but mainly relates to some measure of harm done to hosts by pathogens or the capability of causing disease in host organisms (Read 1994; Casadevall and Pirofski 2001; Thomas and Elkinton 2004). These ideas have been applied to pathogens of various kinds-parasitic, helminthic, bacterial, and viral-infecting a vast number of host types, from plants to nonhuman animals, and humans (Frank 1996; Alizon et al. 2013; Cressler et al. 2016). Classically, it is framed in terms of its relationship to transmission, applying to a suite of traits contributing to a pathogen’s ability to successfully transmit an infection from one host to another (Lipsitch and Moxon 1997; Bull and Lauring 2014).

One of the goals of the evolution of virulence canon is to predict how virulence will change in an evolving interaction between pathogen and host. This is especially relevant in the context of viral pathogens (especially RNA viruses), where the rapid evolution of viruses renders the ecological and evolutionary scales similar (Steinhauer and Holland 1987; Pybus and Rambaut 2009; Duffy 2018). These ideas rose to prominence during the COVID-19 pandemic, in which an array of opinions arose regarding how SARS-CoV-2 populations would evolve with respect to their virulence (Grubaugh et al. 2020; Kissler et al. 2020; van Dorp et al. 2020; Alizon and Sofonea 2021). This pandemic was just the latest manifestation of a long-standing curiosity surrounding the predictive potential of epidemiological methods (Morris et al. 2018; Scarpino and Petri 2019). Several years after the start of the COVID-19 pandemic, questions remain surrounding what rules governed how virulence evolved from the ancestral strain Wu-Hu-1 (Wu et al. 2020) to Alpha (B.1.1.7), Beta (B.1.351), Delta (B.1.617.2) (Lauring and Malani 2021), and Omicron (B.1.1.529) (Fan et al. 2022) variants. Independent of our success in predicting whether evolved variants of interest (VoI) or variants of concern (VoC), the related public health anxiety fortified the idea that we are a long way from formal tools for accurate prediction. Moreover, the reach of these questions is far beyond any one viral outbreak. Alternatively, all of the questions that surfaced during the COVID-19 pandemic apply to many other viral diseases, where we also struggle to make predictions for how pathogens will emerge and how they will evolve once they are present.

Mathematical modeling was foundational in the historical development of epidemiology (Brauer 2017; Siettos and Russo 2013; Kucharski 2020; Jones and Helmreich 2020) and has continued to serve a critical role in the study of infectious outbreaks (Lofgren et al. 2014; Cobey 2020), providing insights for clinical interventions and public health policies (Whitty et al. 2014; Heesterbeek et al. 2015). In addition, models can serve as instruments to explore theoretical questions or to examine how to predict the dynamics of epidemics (Scarpino and Petri 2019). Examining the combined epidemiological and evolutionary dynamics of pathogens present numerous theoretical challenges (Day et al. 2020), and is a frontier of modern epidemiology.

Studies have examined the evolution of multi-strain dynamics (Makau et al. 2022; Kucharski et al. 2016), sometimes framed in terms of “interacting contagions” (Hebert-Dufresne et al. 2020). There have been several large syntheses focused on general questions around virus evolutionary and ecological dynamics (Nowak and May 2000; Weitz 2016). And several scientists have utilized game theoretic methods to model how viruses evolve within particular conditions. One such method is the Price equation approach, employed to reveal various processes influencing evolutionary dynamics (Day et al. 2020). Another related set of methods is associated with the concept of the evolutionary stable strategy (ESS), first pioneered in the study of evolutionary game theory, which describes an optimal, “uninvadable” strategy (Smith and Price 1973; Otto and Day 2007; Smith 1982; Vincent and Brown 2005; Bukkuri and Brown 2021). This perspective has since been applied broadly in infectious contexts, including virus evolution in the setting of different multiplicity of infections (Turner and Chao 1999, 2003) and towards predicting the optimal level of virulence in clinical infections of *Mycobacterium tuberculosis* (Basu and Galvani 2009).

“Evolutionary invasion analysis” as described by Otto and Day (2007) assesses the potential of a population initially fixed for a specific allele to be invaded by a mutant allele that encodes a distinct trait value. In this context, the allele predominant within the population is designated as the *resident allele*, while the emergent allele is denoted as the *mutant allele*. In this study, we apply these methods to examine two highly relevant viral pathogens: SARS-CoV-2 and hepatitis C virus (HCV), each informed by existing real-world empirical data that inform the parameter spaces.

We chose these two study systems because they represent contemporary epidemic scenarios defined by widely different disease ecologies and natural histories. SARS-CoV-2 dynamics are driven by direct transmission between those infected, via both symptomatic and asymptomatic transmission (Mizumoto et al. 2020; Nishiura et al. 2020; Kronbichler et al. 2020). Hepatitis C virus, on the other hand, is largely transmitted indirectly between persons who inject drugs (PWID) in modern settings via drug equipment (Alter 2011).

Using evolutionary invasion analysis, our study offers an integrated method for modeling the evolution of virulence across these two systems. In SARS-CoV-2, we learn that two different conceptual framings of virulence, one involving virulence as a function of the transmission from symptomatic individuals and another where it is a function of both symptomatic and asymptomatic transmission lead to different evolutionary patterns. Examining a mathematical model of HCV in PWID identifies an ESS virulence level that depends on treatment rate, progression into late-stage disease, and self-clearance rate.

Summarizing, we learn that there should be no singular expectation for how virulence will evolve in a population. More broadly, we reflect on our findings for the individual outbreak scenarios (COVID-19 and HCV) and how they may inform larger conversations surrounding how we measure, understand, and prognosticate the evolution of virulence in epidemics, with implications for mathematical epidemiology and public health.

## Methods

### Introduction to Evolutionary Invasion Analysis of Pathogens

To analyze evolutionary invasion, one can follow these steps, which are extracted from prior studies on the topic (Otto and Day 2007; Williams and Kamel 2021):

1. Begin with a model outlining the temporal dynamics of a specific population of interest.
2. Identify the trait under scrutiny and define potential trait values. Additionally, establish a model elucidating the frequency of mutations within the organism’s trait and the dissemination of these mutation variants, either leading to fixation or extinction.
3. Formulate one or more equations delineating the dynamics of a rare mutant allele emerging within a population predominated by a resident allele, commonly termed as “invasion fitness.” Given the rarity of mutations, envision the emergence of mutation-generated phenotypes within an ecological setting near the equilibrium of the resident type.
4. Employ linear stability analyses to address queries regarding the invasion of a novel type. Analyze the fate of a mutant allele by assessing its population growth rate, denoted as invasion fitness, upon its initial introduction as a minor deviation from the stable equilibrium of the population in the absence of the mutant.
5. Derive the requisite condition for the mutant allele to successfully invade the resident population.
6. Explore the evolution of the trait concerning the resident trait value.
7. Identify resident trait values impervious to invasion by any mutant allele, recognized as evolutionary stable strategies (ESSs).

In this investigation, we focus on elucidating the evolutionary dynamics of pathogens within the host population. To achieve this, we employ compartmental epidemiological models, widely recognized for their utility in studying disease dynamics.

Our study investigates how differences in mechanistic relationships between virulence and transmission manifest in evolutionary dynamics. The dynamics of mutant strains can be observed by introducing distinct compartments for infected hosts harboring the mutant variant.

Employing stability analyses of the mutant-free equilibrium enables us to probe the intricacies surrounding the potential invasion of novel pathogen types. These stability conditions serve as pivotal metrics, constituting *fitness functions* for the resident pathogen strain. This framework identifies evolutionary stable strategies as trait values that optimize these *fitness functions*, thus representing configurations resistant to invasion by mutant strains.

Before delving into the detailed discussions, we provide a comprehensive overview of our approach, some of the terminologies employed, and the rationale behind our choice of disease models. We then explain the concept of evolutionary invasion analysis of pathogens, using an elementary susceptible-infected (SI) model. Within the remaining sections of Sect. 2, we construct a generalized framework to analyze the invasion dynamics of mutant strains within host populations existing in equilibrium states with resident strains.

In the next section (Sect. 3), we embark on the development of two distinct epidemiological models tailored for understanding the dynamics of severe acute respiratory syndrome coronavirus 2 (SARS-CoV-2) and Hepatitis C virus (HCV). These models are engineered to capture the interplay between resident and mutant strains of the pathogens within the intricate dynamics of host populations. Leveraging these models, we undertake an analysis of our compartmental epidemiological framework to discern evolutionary stable strategies for the aforementioned pathogens. Through these efforts, we endeavor to examine the evolutionary dynamics in pathogen populations, thereby enriching our understanding of disease transmission dynamics. Before delving into the detailed analysis, we will make a few general notes about this study.

*Notes on the approach* In this study, we examine the impact of pathogen evolution on the parameters of a disease, such as virulence and transmissibility, through a mathematical model with two strains. We utilize a system of ordinary differential equation (ODEs) of the compartmental model to determine the fitness function of the strains. The analysis identifies the fitness function as the $\mathcal {R}_0$, which depends on the evaluation parameters. By establishing the fitness function, we can gain insight into the pathogen’s ESSs and analyze its sensitivity to other parameters. We present an algorithm for these calculations and apply it to evaluating SARS-CoV-2 and HCV using ODE models.

*Notes on terminology* “Strain” is sometimes used in applications of the evolutionary stable strategy in pathogen evolution. In our study, we use the term “strain” to mean different phenotypic variants of a viral pathogen still belonging to the same type. We recognize the dubiousness associated with how viruses are grouped (e.g., clone, population, quasispecies), but are using language that is consistent with others in related fields.

Similarly, “virulence” is a famously complicated term, often used to describe different phenotypic impacts of pathogen infection. For our study, one might use a standard definition related to the harm caused to the host on behalf of a pathogen’s infection. However, because our study utilizes mathematical models, we try to be explicit and consistent about its definitions. We translate virulence as the rate of death from infection (“infected death rate”), as this captures the ultimate sort of harm caused by a pathogen.

The term “fitness function” is utilized with varying contextual meanings. In this instance, we employ it to denote the stability condition for the mutant-free equilibrium within the dynamic system.

*On the choices of disease models* This study focuses on viral pathogens, as understanding and predicting how virulence evolves in these systems have been especially dubious. Dozens of examples could be used to examine this, but we chose two—SARS-CoV-2 and hepatitis C virus (HCV)—both contemporary public health concerns. In addition, they represent diseases with different natural histories, allowing us to examine ESS virulence evolution in many different settings. We emphasize that our goal is not to offer any particular argument or intervention but to examine virulence evolution in varied disease systems.

*Notes on the assumptions* There are a few significant assumptions we will impose to reduce complexity. First, we assume that there are no superinfections with pathogen strains. This means that a host infected with the resident pathogen cannot simultaneously be infected with the invading mutant and that a host immune to the resident pathogen is also immune to the mutant pathogen. Next, we assume that the resident strain is in its endemic equilibrium of the epidemiological compartmental model before the rare mutant appears in the system. Additionally, we assume that general epidemiological compartmental models have infected subsystems with transmission and transition matrices that satisfy the Next-generation matrix theory, as described in Hurford et al. (2010).

Furthermore, we assume that virulence changes due to pathogen mutation, and that the rate of transmission is a function of virulence. This assumption reflects our interest in the evolution of pathogen virulence, and its epidemiological consequences. We should note that, one can readily relax these assumptions and introduce new parameters of interest for their study without altering the analysis steps. That is, the methods applied in this study aren’t specific to any one definition of virulence, or mechanistic relationship between it and other traits.

#### Susceptible-Infected (SI) Model in Evolutionary Invasion Analysis

In this section, we apply evolutionary invasion analysis of pathogens, wherein host population dynamics can be represented using the simple SI modeling approach (Otto and Day 2007, chapter 12.4). This will explain relevant concepts such as the mutant-free equilibrium (MFE), evolutionarily stable strategies (ESSs), the “fitness function”, and the mathematical aspects involved in understanding the fixation or extinction of rare mutant strains. In later sections, we will generalize these concepts for broader applications in pathogen evolution. The results section will discuss the evolutionary invasion analysis of specific pathogen-related diseases.

In this scenario, the ecological model is defined by the dynamics of the susceptible population (*S*) and the infected population (*I*). It is important to note that we are not directly modeling the dynamics of individual pathogens themselves. Instead, we focus on tracking the number of hosts infected by a specific pathogen. We postulate that, in the absence of the mutant strain, the dynamics of the resident pathogen adhere to the SI epidemiological model, with the infected population due to the resident pathogen represented as $I_r$. This is described by Eq. (1), which excludes recovery. Specifically, the fluctuations in the numbers of susceptible and infected individuals are governed by a system of ordinary differential equations (ODEs), expressed as follows:

1 $$\begin{aligned} \begin{aligned} \frac{dS}{dt}&= b -\beta _r S I_r-dS,\\ \frac{dI_r}{dt}&= \beta _r S I_r -(d+\mu _r)I_r. \end{aligned} \end{aligned}$$

The parameter *b* represents the birth/immigration rate of susceptible hosts, while *d* denotes the per capita background mortality rate of hosts. Furthermore, $\mu _r$ signifies the heightened mortality rate induced by the resident strain, which we designate as virulence, and $\beta _r$ denotes the transmission rate. Our focus lies in understanding how virulence and transmission impact the fixation or extinction of a mutant strain. We prioritize virulence ($\mu$) as the determining factor, recognizing that the transmission rate is likely contingent upon virulence, represented as an arbitrary function $\beta (\mu )$. Therefore, the parameter value $\beta _r=\beta (\mu _r)$, where $\beta _r$ is the function value evaluated at $\mu _r$. The precise nature of this function may remain elusive or subject to further investigation. This system exhibits two fixed points $({\hat{S}},{\hat{I}}_r)$: one representing the absence of the pathogen in the population, commonly referred to as the disease-free equilibrium (DFE), denoted as $(\frac{b}{d},0)$, and the other signifying the pathogen’s endemic presence, denoted as $(\frac{d+\mu _r}{\beta _r},\frac{b}{d+\mu _r}-\frac{d}{\beta _r})$. Moreover, the criteria for local stability at the endemic equilibrium can be expressed using the basic reproduction number. This value for the resident strain is denoted by $\mathcal {R}_{0_r}$, and its specific value for this model is given by $\mathcal {R}_{0_r}=\frac{b\beta _r}{d(d+\mu _r)}$. It’s noteworthy that if $\mathcal {R}_{0_r}>1$, then the endemic equilibrium for the resident strain achieves local stability.

The next phase in the evolutionary invasion analysis involves the introduction of a rare mutant allele into a population predominantly characterized by the resident allele. In the context of pathogen evolution, this is achieved by incorporating an infected host population $I_m$ harboring the rare mutant strain. As previously stipulated, we operate under the assumption of no superinfection within the host population. Moreover, we consider the dynamics of the $I_m$ population to mirror those of the $I_r$ population, albeit with distinct parameter values to encapsulate the influence of the mutant pathogen. Specifically, in our case, differing parameter values are allocated for virulence (designated as $\mu _m$ for the mutant strain), consequently affecting the transmission rate $\beta _{m}$. Here, $\beta _m$ represents the transmission function value at $\mu _m$, denoted as $\beta _m=\beta (\mu _m)$. Presently, both the resident and rare mutant strains within the host population adhere to the SI epidemiological model and can be depicted through a system of ODEs, as outlined in Eq. (2):

2 $$\begin{aligned} \begin{aligned} \frac{dS}{dt}&= b -\beta _r S I_r-\beta _m S I_m-dS,\\ \frac{dI_r}{dt}&= \beta _r S I_r -(d+\mu _r)I_r,\\ \frac{dI_m}{dt}&= \beta _m S I_m -(d+\mu _m)I_m. \end{aligned} \end{aligned}$$

*Mutant-free equilibrium, fitness function, and evolutionary stable strategies* It is noteworthy that the system of ODEs presented in Eq. (2) exhibits several equilibrium points, namely the disease-free equilibrium ($I_r=I_m=0$), mutant-strain free equilibrium ($I_m=0$), resident-strain-free equilibrium ($I_r=0$), or the endemic equilibrium with both strains coexisting ($I_r,I_m\ne 0$). In evolutionary invasion analysis, our primary concern is to discern the fate of the mutant allele when introduced as a minor deviation from the stable equilibrium of the population in the absence of the mutant.

We translate this concept to pathogen evolution, utilizing an epidemiological modeling approach, represented as the stability analysis of the MFE. Here, we scrutinize the system’s dynamics around the fixed point wherein only the resident strain is present, with no mutant strain in the host population. We must additionally assume that the ODE model devoid of any host compartment for the mutant strain resides in the endemic equilibrium. In the case of the SI example, this corresponds to the model represented by Eq. (1), with the condition that $\mathcal {R}_{0_r}>1$. If the mutant-free fixed point of the full model (in the case of the SI example, Eq. (2)) is locally stable; even a minor introduction of the infected population carrying the mutant strain will tend to converge towards the mutant-free fixed point. Consequently, this leads to the eventual extinction of the mutant variant. Therefore, the local stability criteria at the MFE can be rearranged to articulate the evolutionary stable strategies for our pertinent decision parameter. In our study of pathogen evolution, we designate this parameter to be virulence.

In the SI epidemiological model, the MFE is given by ${\hat{V}}_2=({\hat{S}},{\hat{I}}_r,{\hat{I}}_m)=(\frac{d+\mu _r}{\beta _r},\frac{b}{d+\mu _r}-\frac{d}{\beta _r},0)$. Additionally, we assume that $\mathcal {R}_{0_r}=\frac{b\beta _r}{d(d+\mu _r)}>1$ to ensure that the resident strain attains an endemic equilibrium within the model described by Eq. (1). Furthermore, the linearized systems of Eq. (2) near the MFE can be represented in matrix form as $\frac{dV}{dt}=J({\hat{V}}_2)V$, where $V=(S\ I_r\ I_m)^T$ and the symbol ‘*T*’ denotes the transpose of the matrix. Here, $J({\hat{V}}_2)$ represents the Jacobian matrix of the system at the MFE ${\hat{V}}_2$, and it is defined as:

3 $$\begin{aligned} \begin{aligned} J({\hat{V}}_2) = \begin{pmatrix} -d-{\hat{I}}_r\beta _r &{} -{\hat{S}}\beta _r &{}-{\hat{S}}\beta _m \\ {\hat{I}}_r\beta _r &{} {\hat{S}}\beta _r -d-\mu _r &{}0 \\ 0 &{} 0 &{}{\hat{S}}\beta _m -d-\mu _m \\ \end{pmatrix}. \end{aligned} \end{aligned}$$

This matrix can be represented as a block diagonal matrix and can be generalized to incorporate additional host population compartments, including multiple infected stages or hosts/reservoirs. In the next section, we will delve deeper into generalizing these concepts. To maintain consistency with these computations, particularly regarding the block form of the matrix, we will employ a more compact matrix notation in the next section.

We have already assumed that $\frac{b\beta _r}{d(d+\mu _r)}>1$ guarantees the local stability of the endemic equilibrium prior to the emergence of the mutant. Consequently, the MFE is locally asymptotically stable if ${\hat{S}}\beta _m -d-\mu _m<0$, and unstable if ${\hat{S}}\beta _m -d-\mu _m>0$. In the latter scenario, the mutant strain with virulence $\mu _m$ invades the resident strain with virulence $\mu _r$. By substituting the value of ${\hat{S}}$, the expression $\mathcal {R}_0({\hat{V}}_2)=\frac{\phi (\mu _m)}{\phi (\mu _r)}$ can be compared with unity to establish a stability criterion for the Mutant-free Equilibrium (MFE). Here, $\phi : \mu \mapsto \frac{\beta (\mu )}{d+\mu }$ represents a function of virulence, where $\mu$ denotes the virulence level. If $\mathcal {R}_0({\hat{V}}_2)>1$, the MFE is locally unstable, while if $\mathcal {R}_0({\hat{V}}_2)<1$, it is locally stable. Thus, the function $\phi$ denotes the fitness of the strain at a given virulence level. Consequently, $\phi$ can be regarded as the “fitness function" for the pathogen.

Now, it is noteworthy that if $\mu ^*=\mathop {\mathrm {arg\,max}}\limits _{\mu } \phi (\mu )$ and $\mu _r=\mu ^*$, then no value of $\mu _m$ can satisfy the condition $\frac{\phi (\mu _m)}{\phi (\mu _r)}>1$. Therefore, the values of $\mu ^*=\mathop {\mathrm {arg\,max}}\limits _{\mu } \phi$ are termed as Evolutionary Stable Strategies (ESSs). The analysis of how the parameters in the function $\phi$ affect the ESSs can be further explored. A detailed discussion of this topic, encompassing general epidemiological models and more specific examples involving pathogens such as SARS-CoV-2 and HCV, will be presented in the subsequent sections.

### Model of Disease Dynamics with the Evolution of Pathogen

**Fig. 1:**
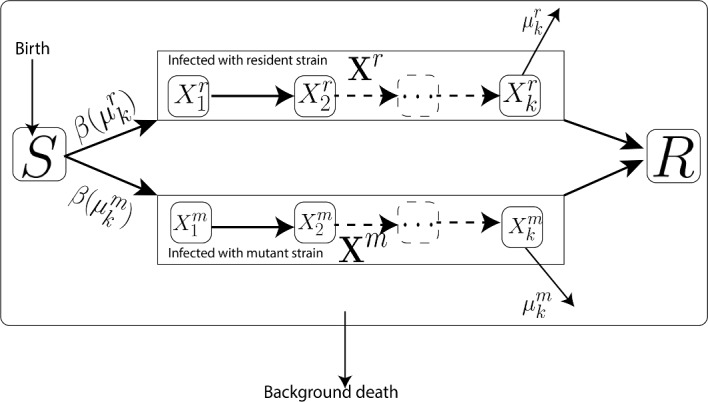
The general compartment model with the assumption of no super-infections. It demonstrates the dynamics of disease with two pathogen strains. Pathogen evolution can impact virulence $\mu _k$ and transmissibility. The transmission rate, represented by $\beta$, is presumed to be a function of the virulence, $\mu _k$

The evolution of communicable disease pathogens can be simulated using a host compartmental ODE model. This section will delve deeper into the ecological modeling of pathogen dynamics. As discussed in the previous section, this can be accomplished by employing epidemiological compartmental models to capture the dynamics of infected hosts. Here, we extend the modeling concepts elucidated in the susceptible-infected (SI) epidemiological models encompass more generalized host compartments, including multiple infected stages or different types of hosts. Here, we analyze a model that involves two viral strains and assumes that a host infected with the resident pathogen (characterized by *r*) cannot simultaneously be infected with the invading mutant (characterized by *m*) and that a host immune to the resident pathogen is also immune to the mutant pathogen (no super-infections). Either strain can contaminate the susceptible compartment (*S*) and then proceed through *k* infected compartments ($\{X_{j}^i\}_{j=1}^k$ for $i\in \{r,m\}$) before reaching the recovery compartment (*R*) (as depicted in Fig. 1). Note that if there are multiple susceptible or recovered compartments, the notations *S* and *R* can be employed to represent the column vectors of those compartments. Furthermore, we will utilize the column vector of infected compartments ${\textbf{X}}_i=(X_1^i\ X_2^i\ldots \ X_k^i)^T$ to denote the infected population with strain $i=r,m$. Similar to the discussion on the Susceptible-Infected (SI) model, we first consider the model with the resident strain, and then introduce the mutant strain by incorporating infected host compartments with mutant strains. To streamline the model notations, we adopt matrix notation in the model equations. Additionally, to facilitate stability analysis using next-generation matrix computations (Diekmann et al. 1990, 2010), we employ specific notations to animate the linearized infected subsystems of the form $\frac{d{\textbf{X}}}{dt}=(\textbf{SF}-{\textbf{D}}){\textbf{X}}$, where ${\textbf{X}}$ represents the infected compartments, $\textbf{SF}$ denotes the transmission matrix (representing all flows from uninfected to infected), and ${\textbf{D}}$ signifies the transition matrix (representing all other flows) (Castillo-Garsow and Castillo-Chavez 2020; Diekmann et al. 2010).

More specifically, the major matrix notations used in the ODE models are explained as follows, with other parameters detailed in Table 1:

- ${\textbf{S}}$: This term refers to a diagonal matrix of susceptibles, adjusted to facilitate the calculation of the transformation from susceptible individuals (*S*) to the infected compartments. This matrix resolves the isolated susceptible population from the transmission matrix $\textbf{SF}$, where ${\textbf{F}}$ represents the transmission parameters in a linearized system.
- $\textbf{SF}_j$: Represents the transmission matrix, whose elements correspond to transmission events resulting in the acquisition of an epidemiological infection ($X_j$). This matrix captures the dynamics of disease transmission within the population (Diekmann et al. 2010).
- ${\textbf{D}}_j$: Denotes a transition matrix capturing all other changes in the disease compartments $X_j$. This matrix encapsulates various processes affecting disease dynamics, such as recovery, mortality, and movement between compartments (Diekmann et al. 2010).
- $\mathcal {S}$: This diagonal matrix with susceptible populations is utilized when multiple host compartments are present in the model. If there is only one susceptible compartment, then $S=\mathcal {S}$.

**Table 1:** This table provides a description of major parameters in generalized epidimiological models (Eqs. (4) and (7))

Notation	Description
$b(N)$	A birth function with respect to a population of size *N* and exclusively contributes to the susceptible compartment
$d(.)$	A natural death function
$f_j$	Transmission rates of the disease with strain *j* for $j=r,\ m$
$g_j$	Recovery rates of the disease with strain *j* for $j=r,\ m$

#### Epidemiological Model for the Resident Strain in the Absence of Mutant Strain

First, we will model the dynamics of the pathogen using the host compartments in the absence of the mutant strain. The dynamics of the resident pathogen adhere to epidemiological models, with the infected population due to the resident pathogen represented as ${\textbf{X}}_r$. This is described by Eq. (4), incorporating the variables and parameters discussed above. The dynamics of the numbers of susceptible and infected individuals with a resident pathogen are governed by a system of ODEs, expressed as follows:

4 $$\begin{aligned} \begin{aligned} \frac{dS}{dt}&= b(N)-\mathcal {S}f_r{\textbf{X}}_r -d(S)\\ \frac{dR}{dt}&=g_r{\textbf{X}}_r-d(R)\\ \frac{d{\textbf{X}}_r}{dt}&= {\textbf{S}}{\textbf{F}}_r{\textbf{X}}_r-{\textbf{D}}_r{\textbf{X}}_r. \end{aligned} \end{aligned}$$

Similar to the discussion on the susceptible-infected (SI) model, this generalized model also possesses two equilibrium points: the DFE and the endemic equilibrium. We utilize the notations $\textbf{S}^{*},\ {\bar{{\textbf{F}}}}_{r},\ {\bar{{\textbf{D}}}}_{r}$ to denote the values of those corresponding matrices at the DFE of Eq. (4). The linearized subsystem of the infection compartments of the model described in Eq. (4) is given by $\frac{d{\textbf{X}}_{r}}{dt}= \big ({\textbf{S}}{\textbf{F}}_{r}-{\textbf{D}}_{r}\big ){\textbf{X}}_r$, and the next-generation matrix can be employed to compute the basic reproduction number ${\mathcal {R}_0}_r$. This concept is summarized in Remark 1.

##### Remark 1

(Basic reproduction number for resident-strain model) Suppose a single-strain model is described by Eq. (4). Then, the basic reproduction number for the resident strain, denoted ${\mathcal {R}_0}_r$, is given by:

5 $$\begin{aligned} \mathcal {R}_{0r} = \rho (\textbf{S}^{*}{\bar{{\textbf{F}}}}_{r},{\bar{{\textbf{D}}}}_{r}^{-1}) \end{aligned}$$

where $\rho (.)$ represents the spectral radius of a given matrix. Furthermore, the DFE of Eq. (4) is locally asymptotically stable if ${\mathcal {R}_0}_r<1$, but unstable if ${\mathcal {R}_0}_r>1$. Note that, the eigenvalues of a block upper triangular matrix of the form,

6 $$\begin{aligned} J(S^*)=\begin{pmatrix} J_* &{} M_r \\ 0 &{} \textbf{S}^*\bar{\textbf{F}}_{r}-{\bar{\textbf{D}}_{r}} \end{pmatrix} \end{aligned}$$

determine the stability condition for the DFE of Eq. (4). Here, $J_*,\ M_r$ are some matrices corresponding to the ODEs of susceptibles and recovered compartments. Therefore, the invasion of the pathogen depends on the sign of the maximum eigenvalue of $\textbf{S}^*\bar{\textbf{F}}_r-{\bar{\textbf{D}}_r}$. This can be expressed in terms of the condition of ${\mathcal {R}_{0}}_{r}$ (a detailed explanation can be found in van den Driessche and Watmough (2002) and Hurford et al. (2010)).

As discussed in Sect. 2.1, we assume that the endemic equilibrium for the resident strain is locally stable in evolutionary invasion analysis. Hence, the maximum eigenvalue of the Jacobian matrix $J_2$ at the endemic equilibrium of the system described in Eq. (4) is negative.

#### Introduction of a Rare Mutant to the Epidemiological Model with Resident Strain

Like Sect. 2.1.1, the next stage of evolutionary invasion analysis involves introducing a rare mutant allele into a population primarily characterized by the resident allele. This is accomplished by integrating an infected host population ${\textbf{X}}_m$ containing the rare mutant strain. As previously stated, we assume no superinfection within the host population. Additionally, we model the dynamics of the ${\textbf{X}}_m$ population to resemble those of the ${\textbf{X}}_r$ population, albeit with distinct parameter values to account for the influence of the mutant pathogen. Specifically, this study assumes that the pathogen’s evolution influences the disease’s virulence and transmission. Additionally, we assume that the transmission rate $\beta$ of fully infected hosts depends on virulence (as shown in Fig. 1) to derive a fitness function for the strains. Now, both the resident and mutant strains within the host population adhere to the general epidemiological model can be expressed as follows (Eq. (7)):

7 $$\begin{aligned} \begin{aligned} \frac{dS}{dt}&= b(N)-\mathcal {S}f_r{\textbf{X}}_r-\mathcal {S} f_m{\textbf{X}}_m -d(S)\\ \frac{dR}{dt}&=g_r{\textbf{X}}_r+ g_m {\textbf{X}}_m-d(R)\\ \frac{d{\textbf{X}}_r}{dt}&= {\textbf{S}}{\textbf{F}}_r{\textbf{X}}_r-{\textbf{D}}_r{\textbf{X}}_r \\ \frac{d{\textbf{X}}_m}{dt}&= {\textbf{S}}{\textbf{F}}_m{\textbf{X}}_m-{\textbf{D}}_m{\textbf{X}}_m \end{aligned} \end{aligned}$$

where the notation is explained in Fig. 1, Table 1, and previous sections.

The principle that a disease-free equilibrium must be established as a requirement for disease models is widely recognized. This is because the transmission of the pathogen cannot occur without any initial cases of infection. Hence, the right-hand side of the infection groups in the general model, as outlined in Eq. (7), has the form *AX*, where *A* is a matrix (whose entries may depend on the infection variables) and *X* is the vector of infection groups. Furthermore, *A* can be decomposed into the ${\textbf{S}}{\textbf{F}}-{\textbf{D}}$ form. To achieve the basic reproduction number, $\mathcal {R}_0$, biologically relevant values for ${\textbf{S}}{{\textbf{SF}}_{j}}$ and ${\textbf{D}_j}$ are chosen, satisfying the hypothesis of next-generation theory (${\textbf{S}}{{\textbf{SF}}_{j}},\ {\textbf{D}_j}^{-1}>0$ and the spectral bound of $-{\textbf{D}_j}$ being less than zero) (Hurford et al. 2010). The premise of “no superinfections” implies that there is no direct interaction between competing strains, which can be further described as each strain’s derivatives (Eq. (7)) being determined exclusively by its own parameters and infection variables, along with the presence of susceptible variables. With these assumptions and explanations presented in Table 1, the system has the potential to exhibit four distinct equilibrium solutions, denoted as ${\hat{V}}_l=({\hat{S}}_l,, {\hat{R}}_l, \hat{{\textbf{X}}}_{r,l},\hat{{\textbf{X}}}_{m,l})$ for $l=1,\dots , 4$ (as outlined in Table 2). The stability criteria for the equilibrium points can be expressed using a form of basic reproduction number. The Remark 1 and Remark 2 explain the basic reproduction number for the single-strain and two-strain ODE models (Eqs. (7) and (4)), respectively. We utilize the notations $\mathbf {S^*},\ {\bar{\textbf{F}}}_j,\ {\bar{\textbf{D}}}_j$ for $j=r,m$ to denote the values of those matrices at the DFE (denoted by ${\hat{V}}_1$).

**Table 2:** A list of equilibrium points in the mathematical model represented by the system of ODEs shown in Eq. (7)

Fixed point	Description	Existence condition
${\hat{V}}_1=(S^*,0,0,0)$	No infections	Always present in the model
${\hat{V}}_2=({\hat{S}}_r,{\hat{R}}_r,\hat{{\textbf{X}}}_r,0)$	Infected only with resident strain. We define this as MFE	$|{\textbf{S}}{\textbf{F}}_r-{\textbf{D}}_r|_{{\hat{V}}_2}=0$
${\hat{V}}_3=({\hat{S}}_m,{\hat{R}}_m,0,\hat{{\textbf{X}}}_m)$	Infected only with mutant strain	$|{\textbf{S}}{\textbf{F}}_m-{\textbf{D}}_m|_{{\hat{V}}_3}=0$
${\hat{V}}_4=({\hat{S}},{\hat{R}},{\textbf{X}}_r^*,{\textbf{X}}_m^*)$	Infected with resident or mutant strain (Co-infected)	$|{\textbf{S}}{\textbf{F}}_r-{\textbf{D}}_r|_{{\hat{V}}4}=0$ and $|{\textbf{S}}{\textbf{F}}_m-{\textbf{D}}_m|_{{\hat{V}}_4}=0$

Understanding the properties of fixed points is essential for understanding the system’s long-term behavior. In this computation, we leverage the fundamental mathematical theorem that states that if *x* is a non-zero vector and $Ax=0$, then the matrix *A* is singular, meaning that its determinant is zero ($|A|=0$)

##### Remark 2

($\mathcal {R}_0$ for Two-Strain Model) Suppose a two-strain model is explained by the Eq. (7). Then, the $\mathcal {R}_0$ for two-strain model is given by:

8 $$\begin{aligned} {\mathcal {R}_0} = \max _{i=r,m}{\rho (\textbf{S}^*\bar{\textbf{F}}_i{\bar{\textbf{D}}}_i^{-1})}. \end{aligned}$$

In this case, the stability of the DFE can be determined by the following Jacobian matrix (block upper triangular form):

9 $$\begin{aligned} J(S^*)=\begin{pmatrix} J_* &{} M_r &{} M_m \\ 0 &{} \mathbf {S^*}{\bar{\textbf{F}}}_r-{\bar{\textbf{D}}_r} &{} 0\\ 0 &{} 0&{} \mathbf {S^*}{\bar{\textbf{F}}}_m-{\bar{\textbf{D}}_m} \end{pmatrix}. \end{aligned}$$

where $J_*,\ M_r, \ M_m$ are some matrices corresponding to the ODEs of susceptibles and recovered compartments. Therefore, DFE is locally asymptotically stable if ${\mathcal {R}_0}<1$, but unstable if ${\mathcal {R}_0}>1$.

Also, note that if a strain labeled as “mutant” is infecting the host population in the absence of the strain labeled as “resident,” then a model similar to Eq. (4), consisting of a system of ODEs, can be utilized to depict the dynamics of only the mutant strain. Furthermore, the basic reproduction number $\mathcal {R}_{0m}$ for the mutant strain in this model is given by:

10 $$\begin{aligned} \begin{aligned} \mathcal {R}_{0m} = \rho (S^*\bar{F}_m{\bar{\textbf{D}}}_m^{-1}). \end{aligned} \end{aligned}$$

### Mutant-Free Equilibrium and Fitness Function

As outlined in Sect. 2.1, one of the major steps in the evolutionary invasion analysis involves assessing the “invasion fitness”. As outlined in Sect. 2.1.1, the “invasion fitness” in pathogen evolution, modeled by epidemiological compartmental models, can be evaluated using linear stability analysis of the MFE. Therefore, in this section, we will delve into the stability analysis of the MFE, denoted by ${\hat{V}}_2$, which can also be regarded as the endemic equilibrium of the host population infected by the resident strain in the full model described in Eq. (7). This analysis will be analogous to the examination of the DFE, considering the mutant as the sole infected compartment while the resident population is treated as uninfected. Hence, the linear stability of the system described in Eq. (7) at the MFE can be effectively analyzed by employing the next-generation matrix theory (Diekmann et al. 1990, 2010) at ${\hat{V}}_2$. This concept can be formally summarized as follows in Lemma1.

#### Lemma 1

(Basic reproduction number for pathogen mutant invasion) Let the model for an infectious disease be represented by the system of ODEs in Eq. (7) and the MFE is given by ${\hat{V}}_2=({\hat{S}}_r,{\hat{R}}_r,\hat{{\textbf{X}}}_r,0)$. Suppose the endemic equilibrium of the compartmental model described in Eq. (4), restricted to the resident strain, is a locally asymptotically stable equilibrium solution of that system. Consider the basic reproduction number for mutant invasion:

11 $$\begin{aligned} {\mathcal {R}_0}({\hat{V}}_2)=\rho ({{\hat{\textbf{S}}}}_r{\bar{\textbf{F}}}_m{\bar{\textbf{D}}}_m^{-1}). \end{aligned}$$

Then the MFE, denoted as ${\hat{V}}_2=({\hat{S}}_r,{\hat{R}}_r,\hat{{\textbf{X}}}_r,0)$ in Eq. (7), is locally asymptotically stable if ${\mathcal {R}_0}({\hat{V}}_2)<1$, and unstable if ${\mathcal {R}_0}({\hat{V}}_2)>1$.

#### Proof

(Outline of the proof) Notice that, Jacobin matrix of the system (Eq. (7)) at ${\hat{V}}_2$ is given by the form:

12 $$\begin{aligned} J({\hat{V}}_2)=\begin{pmatrix} J_2 &{} M_2 \\ 0&{} J_m={{\hat{\textbf{S}}}}_r{\bar{\textbf{F}}}_m-{\bar{\textbf{D}}}_m \end{pmatrix} \end{aligned}$$

where ${{\hat{\textbf{S}}}}_r$ is the value of ${\textbf{S}}$ at MFE and $J_2, M_2$ are some matrices corresponding to the ODEs for compartments of susceptibles, recovered and infected with the resident strain. Accordingly, it is established that the matrix $J({\hat{V}}_2)$ has a block upper triangular form, consisting of sub-matrices $J_2$ and $J_m$. The eigenvalues of $J({\hat{V}}_2)$ are the same as those of $J_2$ and $J_m$ (for example, see Eq. (3)). Now, notice that $({\hat{S}}_r,{\hat{R}}_r,\hat{{\textbf{X}}}_r)$ represents the endemic equilibrium of the resident pathogen in Eq. (4). Furthermore, $J_2$ denotes the Jacobian matrix for Eq. (4) at this fixed point. Assuming the endemic equilibrium for Eq. (4) is asymptotically stable, real parts of all the eigenvalues of $J_2$ are negative. Therefore, the eigenvalues of $J_m$ determine the stability of the MFE, ${\hat{V}}_2$, for Eq. (7). If all the eigenvalues of $J_m$ have a negative real part, then the MFE, ${\hat{V}}_2$, is locally asymptotically stable, and if at least one eigenvalue is positive, then ${\hat{V}}_2$ is an unstable fixed point. According to the next-generation theorem ( (van den Driessche and Watmough 2002; Hurford et al. 2010)), the maximum real part of all the eigenvalues of $J_m = {{\hat{\textbf{S}}}}_r {\bar{\textbf{F}}}_m - {\bar{\textbf{D}}}_m$ is negative if and only if $\rho ({{\hat{\textbf{S}}}}_r {\bar{\textbf{F}}}_m {\bar{\textbf{D}}}_m^{-1}) < 1$. Similarly, the maximum real part of all the eigenvalues of $J_m$ is positive if and only if $\rho ({{\hat{\textbf{S}}}}_r {\bar{\textbf{F}}}_m {\bar{\textbf{D}}}_m^{-1}) < 1$. Therefore, the MFE, denoted as ${\hat{V}}_2 = ({\hat{S}}_r, {\hat{R}}_r, \hat{{\textbf{X}}}_r, 0)$ in Eq. (7), is locally asymptotically stable if ${\mathcal {R}_0}({\hat{V}}_2) < 1$, and unstable if ${\mathcal {R}_0}({\hat{V}}_2) > 1$. $\square$

Consequently, the calculation of ${\mathcal {R}_0}({\hat{V}}_2)$ constitutes the initial step in the process of determining a fitness function for the resident strain. In this process, we aim to demonstrate ${\mathcal {R}_0}({\hat{V}}_2)$ as a ratio of fitness functions of strains. This can be formalized as Proposition 1.

#### Proposition 1

(fitness function) Suppose the infectious disease model is described by the system of differential equations (ODEs) in Eq. (7), and the mutant-free equilibrium (MFE) is represented by ${\hat{V}}_2=({\hat{S}}_r,{\hat{R}}_r,\hat{{\textbf{X}}}_r,0)$. Let’s assume that the endemic equilibrium of the compartmental model defined in Eq. (4), focused on the resident strain, is a locally asymptotically stable equilibrium solution. Then, the basic reproduction number for mutant invasion, denoted by ${\mathcal {R}_0}({\hat{V}}_2)$ and utilized in the stability criteria outlined in Lemma 1, can be evaluated as follows:

13 $$\begin{aligned} \begin{aligned} {\mathcal {R}_0}({\hat{V}}_2)&=\frac{\phi _m}{\phi _r} \end{aligned} \end{aligned}$$

where $\phi _j ={\mathcal {R}_0}_j\prod _{i=2}^n \Big |\frac{\lambda _i({\textbf{E}}^T{\textbf{S}}^*\bar{{\textbf{F}}}_j\bar{{\textbf{D}}}_j^{-1}{\textbf{E}})}{\lambda _i({\textbf{E}}^T{{\hat{\textbf{S}}}}_r\bar{{\textbf{F}}}_j\bar{{\textbf{D}}}_j^{-1}{\textbf{E}})}\Big |$ for $j=r,m$, and ${\textbf{E}}$ is an auxiliary matrix. Here, $\lambda _i(.)$ denotes the $i^{th}$ eigenvalue of a given matrix, and ${\textbf{E}}^T$ represents the transpose of the matrix ${\textbf{E}}$.

#### Outline of the proof

Initially, we leverage the concept of multiplying by an auxiliary matrix to reduce the dimension of the next-generation matrix, as discussed in Diekmann et al. (2010). This process yields a reduced-dimension next-generation matrix containing only non-zero eigenvalues. The goal is to reduce the dimensionality of the next-generation matrices ${\textbf{S}}^*\bar{{\textbf{F}}}_j\bar{{\textbf{D}}}_j^{-1}$ and ${{\hat{\textbf{S}}}}_r\bar{{\textbf{F}}}_j\bar{{\textbf{D}}}_j^{-1}$ for $j=r,m$. Given that only the parameter values of these matrices are changing (with unchanged rows of all zeros), the same auxiliary matrix ${\textbf{E}}$ can be applied in the process outlined in Diekmann et al. (2010). We then utilize two key properties of matrices: the determinant (denoted by $\det (.)$) of a matrix equals the product of its eigenvalues, and the determinant of the product of matrices equals the product of their determinants. Notably, the absolute value of the determinant of the reduced-dimensional next-generation matrices (for $j=r,m$) can be expressed as follows:

14 $$\begin{aligned} \begin{aligned} |\det ({\textbf{E}}^T{\textbf{S}}^*\bar{{\textbf{F}}}_j\bar{{\textbf{D}}}_j^{-1}{\textbf{E}})|&=|\det ({\textbf{E}}^T{\textbf{S}}^*)|\cdot |\det (\bar{{\textbf{F}}}_j\bar{{\textbf{D}}}_j^{-1}{\textbf{E}}) |, \\ |\det ({\textbf{E}}^T{{\hat{\textbf{S}}}}_r\bar{{\textbf{F}}}_j\bar{{\textbf{D}}}_j^{-1}{\textbf{E}})|&=|\det ({\textbf{E}}^T{{\hat{\textbf{S}}}}_r)|\cdot |\det (\bar{{\textbf{F}}}_j\bar{{\textbf{D}}}_j^{-1}{\textbf{E}}) |. \end{aligned} \end{aligned}$$

Now, by taking the ratios of these quantities, we can achieve the following expression:

15 $$\begin{aligned} \frac{|\det ({\textbf{E}}^T{{\hat{\textbf{S}}}}_r\bar{{\textbf{F}}}_m\bar{{\textbf{D}}}_m^{-1}{\textbf{E}})|}{|\det ({\textbf{E}}^T{{\hat{\textbf{S}}}}_r\bar{{\textbf{F}}}_r\bar{{\textbf{D}}}_r^{-1}{\textbf{E}})|} =\frac{|\det ({\textbf{E}}^T{\textbf{S}}^*\bar{{\textbf{F}}}_m\bar{{\textbf{D}}}_m^{-1}{\textbf{E}})|}{|\det ({\textbf{E}}^T{\textbf{S}}^*\bar{{\textbf{F}}}_r\bar{{\textbf{D}}}_r^{-1}{\textbf{E}})|} \end{aligned}$$

Note that the maximum absolute values of eigenvalues of these reduced-dimensional next-generation matrices correspond to the basic reproduction numbers (Diekmann et al. 2010). More specifically, ${\mathcal {R}_0}({\hat{V}}_2)=\rho ({\textbf{E}}^T{{\hat{\textbf{S}}}}_m\bar{{\textbf{F}}}_m\bar{{\textbf{D}}}_m^{-1}{\textbf{E}})$, and ${\mathcal {R}_0}_j=\rho ({\textbf{E}}^T{\textbf{S}}^*\bar{{\textbf{F}}}_j\bar{{\textbf{D}}}_j^{-1}{\textbf{E}})$ for $j=r,m$. Furthermore, notice that $\rho ({\textbf{E}}^T{{\hat{\textbf{S}}}}_m\bar{{\textbf{F}}}_m\bar{{\textbf{D}}}_m^{-1}{\textbf{E}})=1$ (van den Driessche and Watmough 2002; Hurford et al. 2010). Now, using the property that the determinant of a matrix is equal to the product of its eigenvalues and substituting the eigenvalue product into Eq. (15) with the maximum value as the basic reproduction numbers, we can achieve the result. $\square$

The fitness function for the strains can be defined as follows:

16 $$\begin{aligned} \Phi (\mu _k^r,\mu _k^m)= \mathcal {R}_0({\hat{V}}_2) -1, \end{aligned}$$

where the mutant will invade if and only if $\Phi (\mu _k^r,\mu _k^m)>0$. It is worth mentioning that different choices for the fitness function exist, such as $\Phi _1(\mu _k^r,\mu _k^m)=\phi _m-\phi _r$. However, the $\mathcal {R}_{0}$ value for the disease models can be easily obtained in epidemiology, making it a suitable choice for our calculations. Examples will be provided in other sections.

We aim to identify the virulence level that maximizes the invasion’s fitness. In other words, we are interested in finding a strain that a mutant cannot invade. For discussion, we will assume $\mathcal {R}_0({\hat{V}}_2)=\frac{\phi (\mu _k^m)}{\phi (\mu _k^r)}$, which is consistent with practical applications and Eq. (13). Under this assumption, the optimal fitness is achieved when $\mu _k^*=\mathop {\mathrm {arg\,max}}\limits {\phi (\mu _k)}$. This value represents the evolutionary stable strategy (ESS) for the pathogen.

**Fig. 2:**
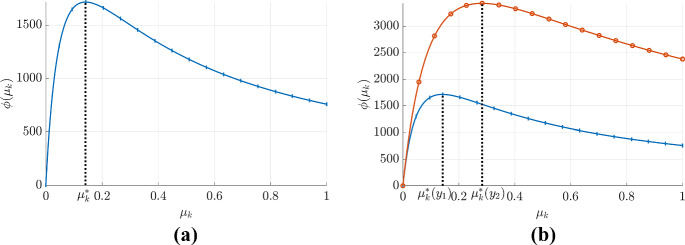
Fitness function and evolutionary stable strategy for a virus population. Panel **a** presents the fitness function ($\phi (\mu )$) as a function of virulence for a given strain, assuming no direct dependence from others. It should be noted that this function exhibits a global maximum, achieved at $\mu _k = \mu _k^*$, indicating $\mu _k^*$ as the ESS for the pathogen. Panel **b** illustrates how the fitness function changes with the parameter $y$. The red (with open circles) and blue curves (with vertical marks) represent the fitness function when the parameter $y$ takes values $y_2$ and $y_1$, respectively. Notably, the global maximum of each of these curves is achieved at $\mu _k^*(y_2)$ (for the red curve, open circles) and $\mu _k^*(y_1)$ (for the blue curve, vertical marks), indicating the ESS for the respective parameter values. This observation illustrates the change in ESS with a given parameter $y$ (Color figure online)

### Evolutionary Stable Strategies

Determining an ESSs for a pathogen is crucial in understanding the dynamics of pathogen populations. The concept of ESS is based on the premise that if a current pathogen population with specific parameters is not vulnerable to invasion by a mutant, then these parameters represent a stable strategy for the pathogen. The fitness function, $\phi (\mu _k)$, is used to analyze these strategies. As previously discussed, the ESS is given by $\mu _k^*=\mathop {\mathrm {arg\,max}}\limits {\phi (\mu _k)}$. This can be demonstrated through Fig. 2a where it can be seen that a pathogen with a virulence value of $\mu _k^r=\mu _k^*$; cannot be invaded by a mutant as its fitness is at its maximum. If the fitness function, $\phi (\mu _k)$, is continuous on the closed interval [0, 1], then we can determine the global ESS by calculating the absolute maximum value.

Moreover, if $\phi (\mu _k)\in C[0,1]$ is twice differentiable at $\mu _k^*$, derivative tests can be utilized to find the local/absolute maximum values and ESS. It can be concluded that if $\phi (\mu _k)$ is twice differentiable at $\mu _k^*$, then $\mu _k^*$ is an ESS if and only if

17 $$\begin{aligned} \frac{d\phi }{d\mu _k}\Big |_{\mu _k=\mu _k^*}&=0,~~~ \text { and } ~~~ \frac{d^2\phi }{d\mu _k^2}\Big |_{\mu _k=\mu _k^*}< 0 . \end{aligned}$$

### Sensitivity Analysis of ESS

After determining the ESS, it is beneficial to analyze the sensitivity of the ESS with regard to other parameters (as illustrated in Fig. 2b). For any given parameter *y*, the change in the ESS $\mu _k^*$ with respect to the increase of *y* can be determined by calculating $\frac{d\mu _k^*}{dy}$. In order to do so, let $\psi (x,y)=\frac{\partial \phi (\mu _k,y)}{\partial \mu _k}\Big |_{\mu _k=x}$ and let $\mu _k^*$ be a function of *y*. It is important to note that $\mu _k^*$ satisfies the following equation:

18 $$\begin{aligned} \frac{\partial }{\partial y}\psi (\mu _k^*,y)= \frac{\partial \psi (\mu _k,y)}{\partial \mu _k}\Big |_{\mu _k=\mu _k^*}\frac{d \mu _k^*}{d y}+ \frac{\partial \psi (\mu _k,y)}{\partial y}\Big |_{\mu _k=\mu _k^*}=0 \end{aligned}$$

and it can be rewritten as:

19 $$\begin{aligned} \frac{d \mu _k^*}{d y} = - \frac{\frac{\partial ^2 \phi (\mu _k,y)}{\partial y \partial \mu _k}\Big |_{\mu _k=\mu _k^*}}{\frac{\partial ^2 \phi (\mu _k,y)}{\partial ^2 \mu _k}\Big |_{\mu _k=\mu _k^*}}\propto \frac{\partial ^2 \phi (\mu _k,y)}{\partial y \partial \mu _k}\Big |_{\mu _k=\mu _k^*}. \end{aligned}$$

Consequently, by examining the sign of the partial derivative of $\phi (\mu _k,y)$ with respect to the parameter *y* at $\mu _k=\mu _k^*$, one can determine the sensitivity of the ESS with respect to the change in *y*. This allows for an analysis of the behavior of the ESS with respect to any given parameter without the need for an explicit calculation of $\mu _k^*$. This calculation can further be simplified with the given problem. For example, let $\phi (\mu _k, y)=\frac{U(\mu _k,y)}{W(\mu _k, y)}$ where $U, W>0$. This results in the following expression:

20 $$\begin{aligned} \frac{\partial ^2 \phi (\mu _k,y)}{\partial y \partial \mu _k}\Big |_{\mu _k=\mu _k^*} \propto \frac{\partial }{\partial y}\big ( W \frac{\partial U}{\partial \mu _k}- U \frac{\partial W}{\partial \mu _k} \big )\Big |_{\mu _k=\mu _k^*} \end{aligned}$$

and, the sign of $\frac{d \mu _k^*}{d y}$ is equal to the sign of $\frac{\partial }{\partial y}\big ( W \frac{\partial U}{\partial \mu _k}- U \frac{\partial W}{\partial \mu _k} \big )\Big |_{\mu _k=\mu _k^*}$.

The calculation of the ESS and the analysis of changes in the ESS with respect to parameters can be summarized in a structured framework. This framework provides a systematic approach for analyzing the stability of ESS and its sensitivity to different parameters, which is crucial in understanding the behavior of the pathogen population.

**Table:** 

Box 1. Framework for calculating ESS and conducting sensitivity analysis	
1. Use the ODE model (SIR, SEIR, etc.) and calculate the basic reproduction number $\mathcal {R}_{0}$. (This is just the mathematical model currently used for the diseases, assuming only one strain for the pathogen.)	
2. Choose a base model parameter (decision variable) defined by evolution. (In this study, we consider the mortality rate due to the disease (virulence) as that parameter.)	
3. You may categorize the model parameters into categories such that the parameters that are	
$\bullet$ independent of the evolution	
$\bullet$ dependent on the evolution but not directly related to the decision variable, or	
$\bullet$ function of the decision variable. (For example, consider the transmission rate $\beta$ as a function of virulence $\mu _k$).	
4. Find MFE, ${\hat{V}}_2$ and evaluate the ${\mathcal {R}_0}({\hat{V}}_2)=\frac{\phi _m}{\phi _r}$.	
5. Considers $\phi (x)=\phi _{x}$ (where *x* depends on the given strain) as a function of the chosen decision variable and finds maxima that provide the ESS.	
6. Finally, consider the equation $\phi (x,y) = \phi _x(y)$ to determine the sensitivity of ESS to the parameter *y*.	

The next section will demonstrate the proposed framework through several examples.

## Results from Illustrative Cases

In this section, we demonstrate the theoretical framework for evaluating an evolutionary stable strategy (ESS), as described in the previous section, using COVID-19 and HCV as case studies.

### SEIR Model Type: SARS-CoV-2 Example

**Fig. 3:**
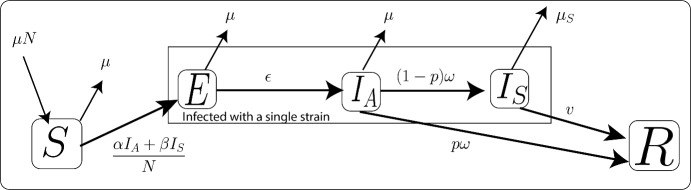
An illustration of the system dynamics of the susceptible, exposed, asymptomatic, symptomatic, and recovered compartments in relation to SARS-CoV-2. Further explanations of the variables and parameters utilized in this figure can be found in Tables 3 and 4. The system of ODEs governing this model is presented in Eq. (21)

We explore the susceptible-exposed-infected-removed (SEIR) compartment model to showcase the proposed theory, with the spread of strains of the SARS-CoV-2 as an example. We use the equations presented in Eq. (21) to model the spread of a specific virus strain in the host population (refer to Ogbunugafor et al. (2020) for details on the single-strain model). Figure 3 visualizes the SEIR model.

21 $$\begin{aligned} \frac{d S}{dt}{} & {} = \mu (N-S) -\big ( \frac{\alpha I_A + \beta I_S}{N}\big )S\nonumber \\ \frac{d E}{dt}{} & {} = \big ( \frac{\alpha I_A + \beta I_S}{N}\big )S-(\epsilon + \mu )E \nonumber \\ \frac{d I_A}{dt}{} & {} = \epsilon E -(\omega + \mu ) I_A\nonumber \\ \frac{d I_S}{dt}{} & {} = (1-p)\omega I_A -(v + \mu _s) I_S\nonumber \\ \frac{d R}{dt}{} & {} = p\omega I_A+v I_S - \mu R \end{aligned}$$

**Table 3:** The variables in Eq. (21)

Variables	Description
*N*	Total population
*S*	Susceptible individuals
*E*	Exposed individuals
$I_A$	Asymptomatic individuals
$I_S$	Symptomatic individuals
*R*	Recovered individuals

All variables are measured as the number of people

**Table 4:** The parameters in Eq. (21)

Parameters	Description	Units
$\mu$	Natural death rate	$\text {day}^{-1}$
$\varvec{\mu _S}$	**Virulence (Infected death rate)**	$\varvec{\text {{\textbf {day}}}^{-1}}$
$\omega ^{-1}$	Expected time in the asymptomatic state	$\text {days}$
*v*	Recovery rate	$\text {day}^{-1}$
*p*	The fraction that moves along the “mild” recovery track	
$\epsilon ^{-1}$	Average number of days before infectious	$\text {days}$
$\alpha$	Transmission rate through the asymptomatic individuals	$\text {day}^{-1}$
$\beta$	Transmission rate through the symptomatic individuals	$\text {day}^{-1}$

The infected death rate is considered as the measure of virulence in the model, and it is highlighted in the table as a row of bold text

Now, consider a two-strain scenario for the SARS-CoV-2 model, where the strains are denoted as *r* (resident) and *m* (mutant). We extend the model by introducing the decision variable $\mu _S$ and assuming that the transmission rate $\beta$ is a function of $\mu _S$, denoted by $\beta _j=\beta (\mu _S^j)$ for $j=r,\ m$. Additionally, we assume that other parameters remain unchanged by evolution. To conform with the notation introduced in the theoretical framework, we use the notation ${\textbf{X}}j=(E_j\ I_{A_j}\ I_{S_j})^T$. In this framing, the matrices ${\textbf{S}}{\textbf{F}}_j$ and $D_j$ relevant to this problem are given by:

$$\begin{aligned} \begin{aligned} {\textbf{S}}{\textbf{F}}_j=\begin{pmatrix} 0 &{}\frac{\alpha S}{N} &{}\frac{\beta _j S}{N}\\ 0&{} 0 &{} 0 \\ 0 &{}0&{} 0 \end{pmatrix}, \ {\textbf{D}}_j=\begin{pmatrix} \epsilon +\mu &{}0 &{}0\\ -\epsilon &{} \omega + \mu &{} 0 \\ 0 &{}-(1-p)\omega &{} v+\mu _S^j \end{pmatrix} \ \text {for} \ j=r,m. \end{aligned} \end{aligned}$$

Now, consider the case of resident strain at the mutant-free equilibrium ${\hat{V}}_r$. It can be observed that $|{\textbf{S}}{\textbf{F}}_r-{\textbf{D}}r|_{{\hat{V}}_r}=0$, indicating that the following expression holds true:

22 $$\begin{aligned} {\hat{S}}_r= \frac{N(\epsilon +\mu )(\omega + \mu )(v+\mu _S^r)}{\epsilon \big (\alpha (v+\mu _s^r)+\beta _r\omega (1-p)\big )}. \end{aligned}$$

Furthermore, it should be noted that the basic reproduction number for the single-strain (*r*) model is given by:

23 $$\begin{aligned} \rho ({\textbf{S}}^*{\bar{\textbf{F}}}_r{\bar{\textbf{D}}}_r^{-1})={\mathcal {R}_0}_r =\frac{S^*\epsilon \big (\alpha (v+\mu _s^r)+\beta _r\omega (1-p)\big )}{N(\epsilon +\mu )(\omega +\mu )(v+\mu _s^r)}. \end{aligned}$$

where $S^*$ is the susceptible population at DFE. Therefore, the basic reproduction number for the MFE of the resident strain can be derived using the following equation:

24 $$\begin{aligned} \rho ({{\hat{\textbf{S}}}}_r{\bar{\textbf{F}}}_m{\bar{\textbf{D}}}_m^{-1}) ={\mathcal {R}_0}({\hat{V}}_2)=\frac{{\hat{S}}_r\epsilon \big (\alpha (v+\mu _s^m) +\beta _r\omega (1-p)\big )}{N(\epsilon +\mu )(\omega +\mu )(v+\mu _s^m)}. \end{aligned}$$

By substituting Eq. (22) into the expression for the basic reproduction number at MFE, we obtain a form:

25 $$\begin{aligned} {\mathcal {R}_0}({\hat{V}}_2)=\frac{\Phi _m}{\Phi _r} \end{aligned}$$

where $\Phi _j={\mathcal {R}_0}_j$. This means that the available basic reproduction number information, modified with the parameters specific to evolution, can be directly used to determine the ESS for models in the SEIR setup. In the event where only $\mu _S$ and $\beta$ parameters are subject to evolution, the expression for ${\mathcal {R}_0}({\hat{V}}_2)$ can be simplified to the form ${\mathcal {R}_0}({\hat{V}}_2)=\frac{\phi (\mu _S^m)}{\phi (\mu _S^r)}$ where the function $\phi (\mu _S)=C_0 + C_1 \frac{\beta (\mu _s)}{v+\mu _s}$, and $C_0=\alpha$ and $C_1=\omega (1-p)$ are constants.

Moreover, the maximum value of $\phi$ with respect to $\mu _s$ may attain at $\mu _S^*$ when the derivative of $\beta$ with respect to $\mu _S$ is equal to $\frac{\beta (\mu _S^*)}{v+\mu _S^*}$:

26 $$\begin{aligned} \frac{d \beta }{d \mu _S} \big |_{\mu _S=\mu _S^*}=\frac{\beta (\mu _S^*)}{v+\mu _S^*}. \end{aligned}$$

It is important to note that if we use the basic reproduction number $\Phi ={\mathcal {R}_0}$ instead of $\phi$ for the analysis, we will obtain the same results since $\Phi =C_2\phi$ with constant $C_2=\frac{S^*\epsilon }{N(\epsilon +\mu )(\omega + \mu )}$. However, to explicitly find the ESSs, we need to model the exact function $\beta (\mu _S)$. In this article, we demonstrate the concept using example functions such as $\beta (\mu _S)=\frac{\mu _S}{a_1+\mu _S},\ \tanh ^2(a_1\mu _S+a_2),\ {{\,\textrm{sech}\,}}^2(a_1\mu _S+a_2)$ and $\sin (a_1\mu _S+a_2)$, where $a_1$ and $a_2$ are constants. (Note that if $\beta$ is a decreasing function, the optimal solution is trivial $\mu _S=0$, so we exclude decreasing functions in this demonstration.) While we do not discuss estimating the $\beta$ function using data in this article, one can easily use any curve-fitting algorithm to identify the transmission function $\beta (\mu _S)$. For example, the theoretical analysis conducted by Massad (1987) utilized data from myxoma viruses to fit the hyperbolic secant squared function to demonstrate the relationship between transmission rate and virulence.

**Fig. 4:**
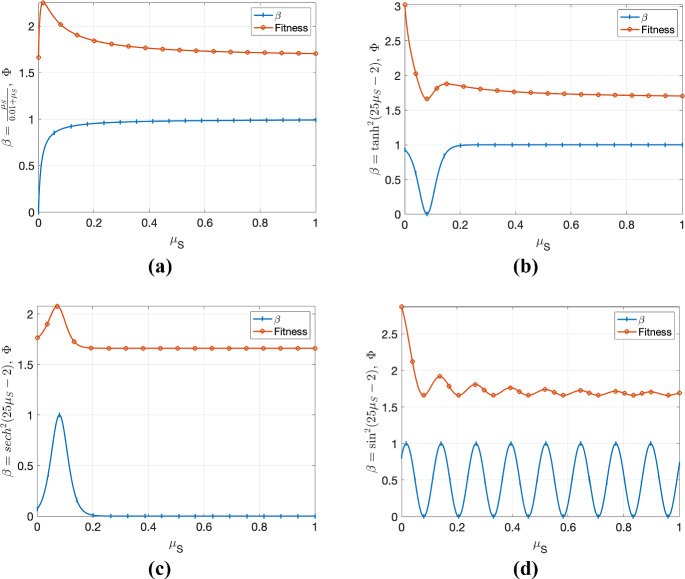
The behavior of the fitness function $\Phi =\mathcal {R}_0$ when transmission function $\beta (\mu _S)$ equals to **a** $\frac{\mu _S}{0.01+\mu _S}$, **b** $\tanh ^2(25\mu _S-2)$, **c** ${{\,\textrm{sech}\,}}^2(25\mu _S-2)$ and **d** $\sin ^2(25\mu _S-2)$. It can be observed that only (**a**) and (**c**) have a non-zero global ESS, while (**b**) and (**d**) have non-zero local ESSs (color figure online)

Depending on the transmission function, there may be one or more local ESSs (see Fig. 4). If $\beta$ is an oscillatory function, the fitness function may also be oscillatory and have multiple local ESS. Since $\mu _S$ is in the closed interval [0, 1] (and assuming that $\beta$ is bounded on [0, 1]), the absolute maximum of the fitness function can be attained at either the endpoints of the interval [0, 1] or at a local maximum of the fitness function. Figure 4 demonstrates the local and absolute maximums of the fitness function, which gives ESS for the pathogen. When a pathogen’s fitness is at the local maximum of its fitness function, it may need a relatively significant change in virulence to attain global ESS. We leave it to the reader to choose the transmission function that best suits their project, and they can expose, extend, and justify their choice using our theoretical basis. For further discussion, we choose $\beta (\mu _S)={{\,\textrm{sech}\,}}^2(a_1\mu _S+a_2)$, but the same analysis can be conducted with any other function.

**Fig. 5:**
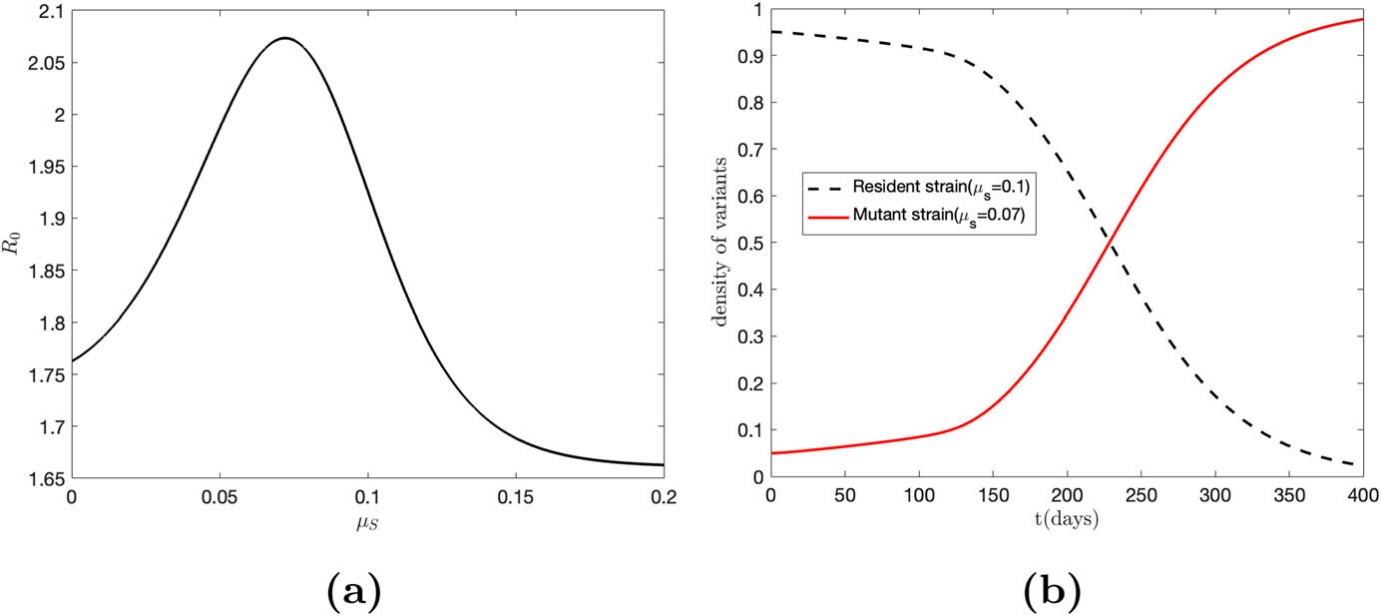
Panel **a** illustrates the basic reproduction number ($\mathcal {R}_{0}$) as a function of virulence ($\mu _S$), considering a transmission rate given by $\beta (\mu _S)={{\,\textrm{sech}\,}}^2(25\mu _S-2)$. The plot reveals a global maximum of $\mathcal {R}_{0}$ occurring around $\mu _S=0.07$. In panel **b**, the red curve represents the infected population with the mutant strain ($\mu _S=0.07$), exhibiting an increasing trend, while the black curve depicts the infected population with the resident strain ($\mu _S=0.1$), showing a decreasing trend. This observation suggests that the mutant variant is invading the population, indicating that the resident strain is not in its ESS (Color figure online)

To illustrate the theoretical concepts discussed earlier in the case of SARS-CoV-2, we utilize the parameter values from Table 4 with the transmission function $\beta ={{\,\textrm{sech}\,}}^2(25\mu _S-2)$. With fixed parameter values taken from Ogbunugafor et al. (2020): $\mu =0.000034$, $\omega ^{-1}=3.119$, $v=0.031$, $p=0.956$, $\epsilon ^{-1}=2.381$, and $\alpha =0.429$. As shown in Fig. 5, panel (a) illustrates the variation of $\mathcal {R}_{0}$ with respect to virulence, while panel (b) displays the host population density for both resident ($\mu _S=0.1$) and mutant strains ($\mu _S=0.07$). Notably, the maximum value of $\mathcal {R}_{0}$ occurs around $\mu _S=0.07$, indicating the dominance of the mutant strain over the resident strain in the long run.

**Fig. 6:**
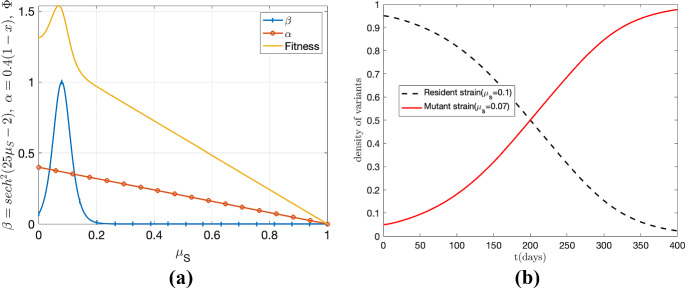
The results incorporate the transmission rate $\alpha (\mu _S)$ with $\beta (\mu _S)={{\,\textrm{sech}\,}}^2(25\mu _S-2)$, using parameters from Fig. 5. Panel **a** shows fitness ($\Phi =\mathcal {R}_{0}$) vs. virulence; $\mathcal {R}_0$ peaks at $\mu _S=0.07$ (orange curve). In panel **b**, the red curve depicts the increasing trend in infected population with the mutant strain ($\mu _S=0.07$), while the black curve shows the decreasing trend in infected population with the resident strain ($\mu _S=0.1$). The dominance of the mutant strain over the resident strain in the long run indicates that the resident strain is not in its evolutionarily stable strategy (ESS) (Color figure online)

To expand on the concept presented earlier, we consider the possibility of incorporating the transmission rate $\alpha$ as a function of virulence. This allows for a more comprehensive analysis of the evolution of virulence in pathogens. To derive the ESS $\mu _S^*$ in this scenario, we take the derivative of the fitness function $\Phi$ with respect to $\mu _S$. The resulting expression is given by:

27 $$\begin{aligned} \alpha '(\mu _S^*)+\frac{(1-p)\omega }{(v+\mu _S^*)^2} \big (\beta '(\mu _S^*)(v+\mu _S^*) -\beta (\mu _S^*)\big )=0 \end{aligned}$$

where prime (^′^) denotes the derivative of the function with respect to $\mu _S$. Figure 6 illustrates the fitness function for the virus and the behavior of the infected host density for each strain when $\alpha (\mu _S)=0.4(1-\mu _S)$ and $\beta (\mu _S)={{\,\textrm{sech}\,}}^2(25\mu _S-2)$. Notably, the global maximum of fitness function occurs around $\mu _S=0.07$. It is observed that the infected population with the mutant strain exhibits an increasing trend, while the infected population with the resident strain shows a decreasing trend (panel (b) of Fig. 6). This observation suggests that the mutant variant (with virulence 0.07) dominates the resident strain (with $\mu _S=0.1$) in the long run, indicating that the resident strain is not in its ESS. Furthermore, we observe that, compared to Fig. 5, the inclusion of $\alpha (\mu _s)$ (in Fig. 6) will decrease the time required for the mutant population to surpass the resident population. The intersection of the mutant and resident curves occurs at $t \approx$ 229 in Fig. 5, while the intersection of these curves occurs at $t \approx$ 200 in Fig. 6. Although we have assumed that only $\beta$ and $\alpha$ are affected by virulence for demonstration purposes, readers can extend this analysis to incorporate other factors.

#### Sensitivity Analysis for SARS-CoV-2

First, we will discuss the sensitivity analysis for the ESS, assuming only $\beta$ is a function of $\mu _S$. Hence, the fitness function $\phi$ can be used for this analysis, providing similar results as analyzing $\Phi ={\mathcal {R}_0}$. We present the analysis using $\phi$ for this case to simplify the calculations. We follow the calculation below to determine the sign of the derivative of ESS with respect to a parameter *y* when the fitness function is $\phi$. Note that:Hence, we can conclude the following result (Eq. (28)) for any parameter *y* with the SARS-CoV-2 model:

28 $$\begin{aligned} \frac{d \mu _S^*}{dy} \propto \frac{\partial }{\partial y}\big (C_1(\frac{d \beta }{d\mu _S}(v+\mu _S)-\beta )\big ) \big |_{\mu _S=\mu _S^*}. \end{aligned}$$

Notice that, the $\mu _S^*$ change only with parameter *v* (if $y=\omega$ or *p*, the right-hand side of Eq. (28) equals zero at $\mu _S^*$ ), and the ESS level of virulence will always increase as the recovery rate increases, regardless of the exact relationship between the transmission rate $\beta$ and virulence $\mu _S$.

Similarly, if we assume both $\beta$ and $\alpha$ as a function of virulence $\mu _S$, relationship in the Eq. (19) can be reduced to,

29 $$\begin{aligned} \frac{d\mu _S^*}{d y} \propto \frac{\partial }{\partial y}\big (C_2(\alpha '(v+\mu _S)^2+(1-p)\omega (\beta '(v+\mu _S)-\beta )\big ) \big |_{\mu _S=\mu _S^*} \end{aligned}$$

where $C_2=\frac{\epsilon }{(\epsilon +\mu )(\omega +\mu )}$. Hence, the changes in ESS $\mu _S^*$ are unaffected by the changes in transition rate $\epsilon$ (from the exposed group to the infected group) and background death rate $\mu$. When considering the recovery rate *v*, we notice that,

$$\begin{aligned} \frac{d \mu _S^*}{d v} \propto \frac{2 \beta (\mu _S^*)}{(v+\mu _S^*)}-\beta '(\mu _S^*). \end{aligned}$$

Hence, ESS $\mu _S^*$ will increase with the recovery rate *v* if and only if $\beta '(\mu _S^*)<\frac{2 \beta (\mu _S^*)}{(v+\mu _S^*)}$.

### Reservoir (W.A.I.T.) Model Type: HCV Example

**Fig. 7:**
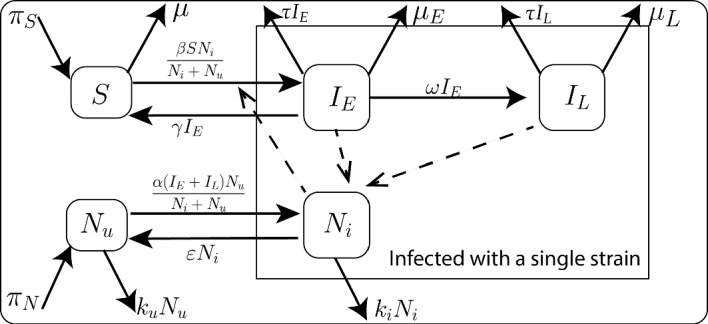
The HCV model is based on a framework for waterborne, abiotic, and indirectly transmitted (W.A.I.T.) disease systems, where an abiotic agent or reservoir (e.g., surface, water supply, or physical instrument) is the primary the vector of transmission (Miller-Dickson et al. 2019; Meszaros et al. 2020). This diagram illustrates the system dynamics of the compartments of HCV. Additional details regarding the variables and parameters employed in this diagram are provided in Tables 5 and 6, respectively. The system of ODEs that governs this model is presented in Eq. (30)

We will perform a similar analysis as the SARS-CoV-2 model to the HCV disease dynamics explained by Eq. (30) and Fig. 7 (using the single-strain model as explained by Miller-Dickson et al. (2019)). Equation (30) is used to model the dynamics of a specific virus strain in host populations and needle compartments.

30 $$\begin{aligned} \begin{aligned} \frac{d S}{dt}&= \pi _S+\gamma I_E-\beta S \frac{N_i}{N_i+N_u}-\mu S \\ \frac{d I_E}{dt}&= \beta S \frac{N_i}{N_i+N_u}-(\omega +\tau +\mu _E+\gamma ) I_E \\ \frac{d I_L}{dt}&= \omega I_E-(\mu _L+\tau )I_L\\ \frac{d N_u}{dt}&= \pi _N-\alpha (I_E+I_L)\frac{N_u}{N_i+N_u}-k_uN_u+\epsilon N_i\\ \frac{d N_i}{dt}&= \alpha (I_E+I_L)\frac{N_u}{N_i+N_u}-k_iN_i-\epsilon N_i \\ \end{aligned} \end{aligned}$$

**Table 5:** The variables in Eq. (30)

Variables	Description
*S*	Susceptible individuals who inject drugs and share needles within the community of people who inject drugs (PWID)
$I_E$	Early-stage infected individuals (acute HCV infection)
$I_L$	Late-stage-infected individuals (chronic HCV infection)
$N_u$	Uninfected needles
$N_i$	Infected needles

All of the variables are measured as the number of people

**Table 6:** The parameters in Eq. (30)

Parameters	Description	Units
$\pi _S$	Birthrate of susceptible	$\text {person}/\text {day}$
$\gamma$	Daily fractional self-clearance rate	$\text {day}^{-1}$
$\mu$	Natural death rate	$\text {day}^{-1}$
$\varvec{\mu _E}$	**Virulence (Infected death rate of early-stage infected population)**	${\textbf{day}}^{-1}$
$\omega$	Transfer rate into late-stage infection	$\text {days}^{-1}$
$\tau$	Rate of entering treatment	$\text {day}^{-1}$
$\pi _N$	Birthrate of uninfected needles	$\text {needles}/\text {day}$
$\epsilon$	Decay rate of HCV infection in needles	$\text {days}^{-1}$
$k_u$	Discard rate of uninfected needles	$\text {day}^{-1}$
$k_i$	Discard rate of infected needles	$\text {day}^{-1}$
$\alpha$	Injection rate times infection of needle probability	$\frac{\text {injections}}{\text {person}.\text {day}}$
$\beta$	Injection rate times infection of host rate	$\frac{\text {injections}}{\text {person}.\text {day}}$
$\mu _L$	Death rate of late-stage infected population	$\text {day}^{-1}$

The infected death rate is considered the measure of virulence in the model, and it is highlighted in the table as a row of bold text

Now, consider a two-strain scenario for the HCV model, where the strains are denoted as *r* (resident) and *m* (mutant). We extend the model by introducing the decision variable $\mu _E$ and assuming that the transmission rate $\beta$ is a function of $\mu _E$, denoted by $\beta _j=\beta (\mu _E^j)$ for $j=r,\ m$. Additionally, we assume that other parameters remain unchanged by evolution. We use the notation ${\textbf{X}}_j=( N_{i_j}\ I_{E_j}\ I_{L_j})^T$ to conform with the notation introduced in the theoretical framework. Here, the matrices ${\textbf{S}}{\textbf{F}}_j$ and ${\textbf{D}}_j$ relevant to this problem are given by (we will use *N* to denote total number of needles in the community):

$$\begin{aligned} \begin{aligned} {\textbf{S}}{\textbf{F}}_j=\begin{pmatrix} 0 &{}\frac{\alpha N_u}{N} &{}\frac{\alpha N_u}{N}\\ \frac{\beta _j S}{N}&{} 0 &{} 0 \\ 0 &{}0&{} 0 \end{pmatrix}, \ {\textbf{D}}_j=\begin{pmatrix} k_i + \epsilon &{}0 &{}0\\ 0 &{} \omega +\tau +\mu _E^j + \gamma &{} 0 \\ 0 &{}-\omega &{} \tau +\mu _L \end{pmatrix} \ \text {for} \ j=r,m. \end{aligned} \end{aligned}$$

We denote the $\bar{S}=\Big (\frac{S}{N}\Big )\Big (\frac{N_u}{N}\Big )$ to simplify the notations carried out in the following calculations. It should be noted that the basic reproduction number for the single-strain (*r*) model is given by:

31 $$\begin{aligned} \rho ({\textbf{S}}^*{\bar{\textbf{F}}}_r{\bar{\textbf{D}}}_r^{-1})={\mathcal {R}_0}_r=\sqrt{\frac{\bar{S}^* \beta _r\alpha (\mu _L+\tau +\omega )}{(\omega +\tau +\mu _E^r+\gamma )(k_i+\epsilon )(\mu _L+\tau )}} \end{aligned}$$

where, $\bar{S}^*$ is the $\bar{S}$ at the DFE. Consider the resident strain at the mutant-free equilibrium ${\hat{V}}_r$. It can be observed that $|{\textbf{S}}{\textbf{F}}_r-{\textbf{D}}r|_{{\hat{V}}_r}=0$, indicating that the following expression holds true:

32 $$\begin{aligned} \hat{\overline{S}}_r=\bar{S}|_{{\hat{V}}_r}=\frac{(\omega +\tau +\mu _E^r+\gamma )(k_i+\epsilon )(\mu _L+\tau )}{ \beta _r\alpha (\mu _L+\tau +\omega )} \end{aligned}$$

Therefore, the basic reproduction number for the MFE of the resident strain can be derived using the following equation:

33 $$\begin{aligned} \rho (\hat{{\textbf{S}}}_r{\bar{\textbf{F}}}_m{\bar{\textbf{D}}}_m^{-1})={\mathcal {R}_0}({\hat{V}}_2) =\sqrt{\frac{\hat{\overline{S}}\beta _m\alpha (\mu _L+\tau +\omega )}{(\omega +\tau +\mu _E^m+\gamma )(k_i+\epsilon )(\mu _L+\tau )}} \end{aligned}$$

By substituting Eq. (32) into the expression for the basic reproduction number at MFE (Eq. (33)), we obtain a form:

34 $$\begin{aligned} {\mathcal {R}_0}({\hat{V}}_2)=\frac{\Phi _m}{\Phi _r} \end{aligned}$$

where $\Phi _j={\mathcal {R}_0}_j$. This means that the available basic reproduction number information, modified with the parameters specific to evolution, may be directly used to determine the ESS in more complex models with reservoirs. If only $\mu _E$ and $\beta$ parameters are subject to evolution, the expression for ${\mathcal {R}_0}({\hat{V}}_2)$ can be simplified to the form ${\mathcal {R}_0}({\hat{V}}_2)=\sqrt{\frac{\phi (\mu _E^m)}{\phi (\mu _E^r)}}$ where the function $\phi (\mu _E)=\frac{\beta (\mu _E)}{\omega +\tau +\mu _E+\gamma }$. In this example, we assume $\mu _E$ is the decision variable, and $\beta$ is a function of $\mu _E$. Since $\mathop {\mathrm {arg\,max}}\limits \sqrt{\phi (x)}=\mathop {\mathrm {arg\,max}}\limits \phi (x)$, ESS can be obtained by analyzing $\phi (\mu _E)$ and we will consider it as the fitness function for the strain. Hence, ESS, denoted $\mu _E^*$, is given by,

35 $$\begin{aligned} \beta '(\mu _E^*)=\frac{\beta (\mu _E^*)}{\omega +\tau +\mu _E+\gamma }, \end{aligned}$$

where prime (^′^) denote the derivative with respect to $\mu _E$.

#### Sensitivity Analysis for HCV

In a similar calculation to the SARS-CoV-2 model example, a derivative of ESS with respect to a given parameter *y* can be explained by,

36 $$\begin{aligned} \frac{d \mu _E^*}{d y} \propto \frac{\partial }{\partial y}\big (\beta '(\omega +\tau +\mu _E+\gamma )-\beta \big ) \big |_{\mu _E=\mu _E^*}. \end{aligned}$$

Hence, ESS is only sensitive to the parameters $\omega ,\ \tau , \gamma$, and $\beta$. Furthermore, it proves that $\frac{d \mu _E^*}{d y}>0$ with parameters $y=\omega ,\ \tau$ or $\gamma$. Therefore, we can conclude that the ESS level of virulence will increase as;

- The treatment rate $\tau$,
- The transfer rate into late-stage infection $\omega$, or
- The self-clearance rate $\gamma$

increases. In addition, when $y=\beta$, the Eq. (36) can be reduced to,

37 $$\begin{aligned} \frac{d \mu _E^*}{d \beta } \propto \big ( (\omega +\tau +\mu _E+\gamma )\frac{\beta ''(\mu _E^*)}{\beta '(\mu _E^*)}-1)\big )<0 \end{aligned}$$

where, (^′′^) denotes the second derivative with respect to the $\mu _E$. Since the maximum of the fitness function has been attained at $\mu _E^*$, the second derivative condition can be reduced to $\beta ''(\mu _E^*)<0$. Therefore, $\frac{d \mu _E^*}{d \beta }<0$, and the ESS level of virulence will decrease as the infection of host rate $\beta$ increases.

## Discussion

In this study, we utilize mathematical approaches to identify the evolutionary stable strategy (ESS) level of virulence for virus pathogens of differing structures and natural history: SARS-CoV-2 and HCV. The viruses underlying these outbreaks exhibit distinct characteristics in the diseases they cause, their modes of transmission, biological structures, and the level of virulence exerted on their hosts. We build models of each, with parameters determined from existing studies that implement published data (Ogbunugafor et al. 2020; Miller-Dickson et al. 2019). Furthermore, we propose a framework for identifying evolutionary stable strategies based on the identification of a fitness function that aids in the invasion analysis of mutant strains. We apply the invasion analysis developed in other texts (Otto and Day 2007) to compute ESS virulence in different viral natural histories. Table 7 summarizes select findings and briefly discusses some of their public health implications.

**Table 7:** Brief summary of ESS virulence changes in different disease models and their public health implications

Model type	ESS virulence increases	ESS virulence decrease	Public health implications
Indirectly transmitted infection (simplified HCV model)	ESS virulence increases as treatment rate, rate of late-stage progression, and self-clearance rate increase	ESS virulence decreases as the transmission rate in hosts increases	Under certain conditions, viruses can evolve increases virulence in populations of individuals with higher rates of self-clearance of a virus
Directly transmitted model (simplified SARS-COV-2 model)	When virulence is a function of transmission from symptomatic individuals, virulence increases as a function of recovery rate	When transmission from both symptomatic and asymptomatic individuals is related to virulence, virulence decreases as the recovery rate increases, while considering other parameters for regularization	Whether asymptomatic and/or symptomatic individuals are transmitting has profound implications for the evolutionary trajectory of traits that confer virulence

In SARS-CoV-2, we examine two different cases:(i) one in which virulence is a function solely of the $\beta$ transmission parameter, where symptomatic individuals transmit to susceptible individuals, and (ii) another where virulence is a function of terms associated with both symptomatic ($\beta$) and asymptomatic ($\alpha$) individuals (See Eqs. (28) and (29)). This duality recapitulates debates early in the COVID-19 pandemic, where experts sought to identify the role of asymptomatic infection in disease dynamics (Moghadas et al. 2020; Mizumoto et al. 2020; Nishiura et al. 2020; Kronbichler et al. 2020; Rothe et al. 2020). Our findings highlight why properly characterizing the transmission mechanism of an emerging infectious disease is so crucial: they offer profoundly different predictions for how ESS virulence will evolve.

When virulence is a function of transmission from symptomatic individuals, the ESS level of virulence increases as a function of recovery rate. In this grim hypothetical scenario, virulence increases as the recovery rate goes up, suggesting that treatments and public health interventions will foster increased virulence. Encouragingly (from a public health perspective), SARS-COV-2 is now widely understood to have more complicated transmission dynamics, with both symptomatic and asymptomatic transmission playing a role (Mizumoto et al. 2020; Nishiura et al. 2020; Kronbichler et al. 2020). Given this natural history, our observations and predictions for optimal (ESS) virulence are more complicated, with the direction of virulence evolution depending on several other mathematical relationships. For example, ESS virulence depends on the slope of the transmission rate satisfying a very particular set of conditions, including the asymptomatic recovery rate. These findings in SARS-CoV-2 highlight the complexity of phenotypic evolution in emerging pathogens: simplistic or narrow views of SARS-CoV-2 natural history are likely inadequate, miss the many nuances and dependencies that define how a given population of viruses will evolve in a population of hosts, and implore much more careful definitions and examinations of virulence.

We also compute the ESS virulence for HCV transmission in a population of persons who inject drugs (PWID). The model of HCV transmission includes an indirect transmission compartment, where infected needles circulate in a population. Using an existing model of HCV dynamics, we observe that the ESS virulence will increase as the treatment rate, rate of movement into the late-stage infection increases, and the self-clearance rate increases. Despite being a public health concern, HCV has highly effective treatments available on the market (Liang and Ghany 2013; Morozov and Lagaye 2018; Hu et al. 2020). Intriguingly, self-clearance rate of HCV is known to be influenced by host genetics, with alleles fostering increased or impaired rates of clearance (Thomas et al. 2009; Prokunina-Olsson et al. 2013; Ge et al. 2009). Our findings highlight why one might expect that virulence evolution may increase in populations of individuals who carry the high-clearance allele under certain conditions. This is an intriguing finding because of what it says about how heterogeneity in host characteristics may influence the trajectory of virus evolution. This finding has been discussed in other settings (Hebert-Dufresne et al. 2020). Further, the HCV model highlights how diseases with an indirect transmission route differ from directly-transmitted pathogens. Future studies in this realm will examine ESS virulence evolution in epidemics with other structures-waterborne, vector-borne, and classical fomite transmission.

This study is not designed to explain any particular outcome or directly inform public health interventions. However, we acknowledge that using well-known human pathogens as examples comes with the risk of misinterpretation. Therefore, we emphasize (rather strongly) that this study only aims to utilize models with good existing data to make a general point about the capriciousness of virulence evolution, as it strongly depends on several features of the virus’s natural history and particulars of transmission. We feel that this is an important point to make in light of misinterpretations of modern findings in infectious diseases, some of which even fuel misinformation.

Moreover, these points transcend any particular virus-host system: we believe that attempts to understand any pathogen-host system will be colored by similar nuances. Furthermore, this study utilizes analytical approaches to study disease dynamics, which are increasingly understood to be defined by nuances that undermine the assumptions of SEIR-style analytical descriptions. While this problem plagues many areas of infectious disease modeling, we acknowledge that it undermines the realism of these simulations. Nonetheless, mathematical modeling remains an important tool for studying disease dynamics because it provides a transparent means to engage the actors that drive infectious outbreaks. Future investigations can utilize other computational approaches for modeling infectious diseases (Marshall and Galea 2015; Cardenas et al. 2022), and examine a growing number of virus (and other pathogen) evolution scenarios.

Our study hopes to add to a growing chorus to refine applications of the evolution of virulence, a canon in evolutionary theory that has helped spawn an entire subfield at the intersection of evolutionary biology and epidemiology (Ebert and Bull 2003). While it has helped to revolutionize our understanding of infectious diseases by offering an evolutionary lens on the host-pathogen interaction, it can sometimes oversimplify how pathogen evolution manifests. Our study focuses on outbreaks caused by single-stranded RNA viruses and still offers diverse patterns in evolutionary outcomes. In response to these (and other) findings, those interested in pathogen evolution should more carefully consider their definition of virulence, what aspects of the disease’s natural history underlie it, and how it may evolve in a given setting. Even more, our findings are consistent with discussions in the broader fields of ecology where experts continue to examine the meaning and consequences of context-dependence (Catford et al. 2022; Hite and de Roos 2023).

The results have several practical implications. For one, attempts to transform disease emergence and epidemiology into predictive sciences must not rely on simplistic assumptions about the relationships between biological traits that drive transmission or confer clinical symptoms. Alternatively, we should appreciate how contexts profoundly shape how complex biological systems function—ideas that have been well-articulated in other areas of disease evolution and ecology. Further, our results highlight why we need continued research into the basic biology involved in infectious disease. In the future, mathematical modeling approaches can implement more detailed findings from natural settings and utilize newer technologies (e.g., artificial intelligence) to better integrate data and understandings of different sorts into responsible and transparent predictive models in epidemiology.

## Data Availability

Code can be found at https://github.com/OgPlexus/ESSvirulence1. Parameter values in the models can be found in the cited references.
